# Neural activity regulates autoimmune diseases through the gateway reflex

**DOI:** 10.1186/s42234-019-0030-2

**Published:** 2019-08-20

**Authors:** Andrea Stofkova, Masaaki Murakami

**Affiliations:** 1 Department of Physiology, Third Faculty of Medicine, Charles University, Prague, Czech Republic; 2 Division of Molecular Psychoimmunology, Institute for Genetic Medicine, Hokkaido University, Kita-15, Nishi-7, Kita-ku, Sapporo, 060-0815 Japan

## Abstract

The brain, spinal cord and retina are protected from blood-borne compounds by the blood-brain barrier (BBB), blood-spinal cord barrier (BSCB) and blood-retina barrier (BRB) respectively, which create a physical interface that tightly controls molecular and cellular transport. The mechanical and functional integrity of these unique structures between blood vessels and nervous tissues is critical for maintaining organ homeostasis. To preserve the stability of these barriers, interplay between constituent barrier cells, such as vascular endothelial cells, pericytes, glial cells and neurons, is required. When any of these cells are defective, the barrier can fail, allowing blood-borne compounds to encroach neural tissues and cause neuropathologies. Autoimmune diseases of the central nervous system (CNS) and retina are characterized by barrier disruption and the infiltration of activated immune cells. Here we review our recent findings on the role of neural activity in the regulation of these barriers at the vascular endothelial cell level in the promotion of or protection against the development of autoimmune diseases. We suggest nervous system reflexes, which we named gateway reflexes, are fundamentally involved in these diseases. Although their reflex arcs are not completely understood, we identified the activation of specific sensory neurons or receptor cells to which barrier endothelial cells respond as effectors that regulate gateways for immune cells to enter the nervous tissue. We explain this novel mechanism and describe its role in neuroinflammatory conditions, including models of multiple sclerosis and posterior autoimmune uveitis.

## Introduction

The nervous system, with its central and peripheral divisions, is the major integration and command center for controlling all other body systems and organs. It processes and transmits information from the internal and external environments to produce an instant response in various effector organs. The neural activity is organized into reflex arcs, which are the basic functional units of the nervous system and composed of (1) sensory receptors, which monitor the environment, (2) afferent nerve fibers, which transmit the monitoring signals to (3) integrating centers, such as central nervous system (CNS) nuclei, and (4) activated efferent nerve fibers, which regulate organ homeostasis (Levine 2007).

All homeostatic reactions in the body depend on nervous reflexes and/or humoral factors including hormones, nutrients, metabolites, and ions, which are at the same time regulated by the neural system. However, unlike humoral factors, electrical impulses running through neural reflex arcs enable instantaneous responses to inner and outer changes. Thus, nervous reflexes are key to survival and homeostasis (Kotas and Medzhitov 2015).

The presence of various neuronal receptors (e.g. for neurotransmitters, growth factors, etc.) on immunocompetent cells implies that neural reflexes also directly control homeostasis of the immune system. In this context, the inflammatory reflex by which the vagus nerve triggers an immunosuppressive signal to reduce an inflammatory response has been described (Andersson and Tracey 2012; Tracey 2016; Pavlov and Tracey 2017). In the case of autoimmune diseases that result from an abnormal immune response against self-tissue, immune homeostasis fails, and nervous reflexes including the inflammatory reflex regulate the disease pathogenesis. A newly discovered mechanism for this regulation is the gateway reflex, which occurs at blood-nervous tissue barriers such as the blood-brain barrier (BBB), blood-spinal cord barrier (BSCB) and blood-retina barrier (BRB). In gateway reflexes, a common effector non-immune cell type is the barrier endothelial cell in the brain, spinal cord or retina and is activated by noradrenergic efferent pathways (Arima et al. 2012, 2015, 2017; Stofkova et al. 2019).

In this review, we first present an overview of fundamental communication networks between neural pathways and barrier endothelial cells with regard to the development and maintenance of the blood-nervous tissue barrier. Second, we discuss a group of gateway reflexes that take advantage of the neuron-endothelial cell communications to open or close immune cell gateways across the BBB, BSCB and BRB at specific vessels of the CNS and retina.

## Neural-endothelial crosstalk

The first recognition that the phenotype of BBB endothelial cells is not intrinsic to these cells but acquired from the neural microenvironment comes from a study performed by Stewart and Wiley (1981). The transplantation of nonvascular tissue from embryonic quail brain into the gut of chick embryos caused the newly formed vascular endothelial cells originating from the host’s gut to develop BBB characteristics. In contrast, the transplantation of embryonic quail mesodermal tissue to embryonic chick brain caused brain vessels vascularizing the grafted mesoderm to not acquire structural or functional barrier features. Follow-up studies focused on identifying the cells in the neural microenvironment that contribute to the development and maintenance of the barrier phenotype. Due to the anatomical proximity of astrocytes to CNS endothelial cells, the earliest of these studies were performed on astrocytes during the induction of BBB properties by endothelial cells. Grafted astrocytes as well as Müller cells to the anterior eye chamber were able to induce barrier characteristics to non-neural vascular endothelial cells (Janzer and Raff 1987; Tout et al. 1993). Consistent with these observations, in vitro investigations showed the importance of astrocytic endfeet for the formation of the BBB (Hayashi et al. 1997). That study used a heterologous co-culture system, in which rat fetal brain astrocytes were cultured on one surface of a porous membrane and human umbilical vein endothelial cells on the opposite surface, enabling the contact of endothelial cells with only astrocytic endfeet projections. This unique culture system revealed the development of barrier activity against inulin and an upregulation of BBB-specific markers, including transferrin receptor, P-glycoprotein, brain-type glucose transporter GLUT-1 and gamma-glutamyltranspeptidase (GGTP) on umbilical vein endothelial cells. Other studies have also proven the contribution of astrocytes to the barrier phenotype and the importance of astrocyte-derived factors on the functional characteristics of BBB endothelial cells (Haseloff et al. 2005; Abbott et al. 2006; Broux et al. 2015).

Other cell types also contribute to the characteristics of barrier endothelial cells, including neurons, pericytes, vascular smooth muscle cells (VSMCs), microglia and oligodendrocytes (Broux et al. 2015; Keaney and Campbell 2015; O’Brown et al. 2018; Miyamoto et al. 2014). In addition, one study suggested that neurons have predominant effects on the acquisition of barrier phenotypes by vascular endothelial cells (Tontsch and Bauer 1991). Here, the co-culture of cerebral capillary endothelial cells with astrocytes, glioma cells and cortical neurons showed that, although both neurons and glial cells induced the activity of GGTP, an enzymatic marker of the BBB, in cerebral capillary endothelial cells, the GGTP activity was higher in endothelial cell co-cultures with neurons or neuronal plasma membranes. Moreover, in early development of the CNS, neurons play a critical role in the BBB formation by cerebral endothelial cells.

Finally, it was reported that the invasion of blood vessels in the mouse developing neural tube occurs at embryonic day (E) 10, which is consistent with neurogenesis starting between E11-E17. At this stage, the newly formed cerebral microvasculature is surrounded by neuroblasts and undifferentiated neurons, and gliogenesis occurs after functional BBB formation, excluding the role of glia cells on BBB development in vivo (Saunders et al. 1999; De Bock et al. 2014; O’Brown et al. 2018). Together, the above studies suggest that neural signals are essential to establishing the barrier function of vascular endothelial cells in the CNS.

Nevertheless, little is known about the cellular and molecular mechanisms for the communication between neurons and endothelial cells that establishes the BBB properties of the CNS endothelium. Given the arrangement of neurons, glial cells and the CNS vasculature, which are collectively referred to as the neurovascular unit (NVU), neurons are not in direct contact with endothelial cells (Iadecola 2017; Kisler et al. 2017). At the level of CNS-penetrating arteries or arterioles, endothelial cells are covered by the extracellular basement membrane and ringed by VSMCs, which are surrounded by astrocytes. At the capillary level, endothelial cells share a common basement membrane with pericytes that are covered by astrocyte endfeet. In this assembly, neurons usually form physical contacts with VSMCs, pericytes and astrocytes, but not directly with endothelial cells (Iadecola 2017; Kisler et al. 2017). However, neurons can communicate with endothelial cells by contact-independent strategies through a wide range of regulatory molecules including growth factors, neurotransmitters, neuropeptides, semaphorins and immunomodulating factors (Chavarría and Cárdenas 2013). Much work has been done on the role of specific neurotransmitters and neuropeptides in neurovascular coupling, a process by which local neuronal activity controls blood flow in the CNS. There are several excellent reviews on this subject (e.g. Drake and Iadecola 2007; McConnell et al. 2017; Iadecola 2017; Kisler et al. 2017). Here we focus on neuronal mechanisms that regulate the barrier phenotype in vascular endothelial cells in the CNS and retina.

## Role of noradrenergic signaling in modulating barrier endothelium

The catecholamines norepinephrine (NE) and epinephrine (EPI) are neurotransmitters widely distributed throughout the CNS and play a role in CNS development, motor control, emotion, motivation, learning, memory formation and processing, as well as neuroendocrine and autonomic regulations (Kobayashi 2001). Their receptors, alpha(α)- and beta(β)-adrenoceptors (ARs), are present on vascular endothelial cells in the brain, spinal cord and retina (Hertz et al. 2010; Böhmer et al. 2014; Haselton et al. 1998).

The central noradrenergic system significantly contributes to the development of the cerebral vasculature, including angiogenesis and the contractile function of blood vessels (Buchholz et al. 2007). NE in the CNS regulates BBB permeability of the vascular endothelium. NE injection to the right lateral cerebral ventricle has been shown to increase BBB permeability for sodium fluorescein dose-dependently, most likely due to the NE-induced pinocytotic activity of endothelial cells via α-ARs (Sarmento et al. 1991). Similar observations have been reported when the locus coeruleus, a major source of NE in the brain, was electrically stimulated. Specifically, BBB permeability was increased in a stimulation frequency-dependent manner, and this effect was completely blocked by the α-AR antagonist phenoxybenzamine, but not by the β-AR antagonist pindolol (Sarmento et al. 1994). In addition, both NE- and EPI-induced leakage of the BBB has been confirmed using an in vitro BBB system composed of brain microvessel endothelial cells (Borges et al. 1994). In that study, the increased monolayer permeability was shown to be mediated by α-ARs, while the activation of β-ARs was observed to decrease monolayer permeability. Thus, these findings demonstrated opposite effects of ARs on BBB functions: β-adrenergic innervation appears to support BBB integrity, whereas α-adrenergic innervation is associated with increased BBB permeability.

In the context of multiple sclerosis (MS), disturbances in the noradrenergic system in the CNS have been described by several studies. However, controversies concerning CNS NE levels in patients with MS still exist. For example, Barkhatova et al. (1998) reported increased NE levels in the cerebrospinal fluid (CSF) of both relapsing-remitting and progressive MS patients compared to controls. On the other hand, Markianos et al. (2009) observed a negative correlation of the CSF level of methoxyhydroxyphenylglycol (MHPG), the major metabolite of NE, with the duration of the illness as well as with the number of relapses, possibly indicating diminishing central noradrenergic system activity with relapses. Moreover, autopsied brains from MS patients showed a reduction in NE levels and astrocyte activation, along with a hypertrophy of tyrosine hydroxylase positive neuronal cell bodies in the locus coeruleus compared to controls (Polak et al. 2011).

Experimental autoimmune encephalomyelitis (EAE), an animal model of MS, is an invaluable tool for studying the pathogenesis of the BBB breakdown and the infiltration of immune cells into the CNS. Many different models of EAE have been developed based on the animal species, encephalitogenic antigen and technique used to induce the disease. One of the most popular murine models is EAE induced by a myelin-derived antigen, myelin oligodendrocyte glycoprotein 35–55 (MOG_35–55_), which is given emulsified in complete Freund’s adjuvant (CFA) as a subcutaneous injection. The adoptive transfer of MOG-specific CD4+ T cells (pathogenic CD4+ T cells) from donor mice that were actively immunized by MOG to naïve recipient mice can also induce EAE. This adoptive transfer EAE model allows study of the autoimmune CNS inflammation induced specifically by pathogenic CD4+ T cell responses (Glatigny and Bettelli 2018; Rangachari and Kuchroo 2013; Tanaka et al. 2017a). Indeed, we showed that the MOG-tetramer+ cells in pathogenic CD4+ T cells, which are in vitro restimulated splenic CD4+ T cells isolated from active EAE mice (direct immunization of a MOG peptide in wild type mice), are about 20% just before the transfer to wild type mice (Arima et al. 2017). On the other hand, pathogenic CD4+ T cells prepared from MOG-specific TCR transgenic mice (2D2 mice) are not MOG-tetramer+ cells (Sabatino et al. 2011). Therefore, we hypothesized that the majority of our pathogenic CD4+ T cells that survived even after in vitro restimulation are MOG-specific regardless of the MOG-tetramer staining.

EAE models support clinical findings of altered NE and EPI levels in patients with MS. Reduced cortical and spinal cord NE levels as well as neuronal damage and astrocyte activation in the locus coeruleus have been reported in EAE mice (Polak et al. 2011). In the lumbar or lumbosacral spinal cord, the decreased NE levels in EAE have been confirmed by other groups as well (Krenger et al. 1986, 1989; Musgrave et al. 2011). On the other hand, in the craniothoracal region of the spinal cord, NE levels decreased only during attacks of chronically relapsing allergic encephalomyelitis and returned to normal in remission (Krenger et al. 1986). Regarding the brainstem of mouse EAE, NE levels increased (Musgrave et al. 2011), but in rat EAE they decreased (Krenger et al. 1989). Despite this discrepancy among studies, which might be due to differences in species or experimental settings, these results point to an altered central noradrenergic system in MS and related animal models. The importance of these pathologic alterations of the noradrenergic system for EAE development has been demonstrated either by increasing the synthesis of NE in the CNS (Simonini et al. 2010; Li et al. 2018) or by treatment with AR antagonists (Arima et al. 2012; Brosnan et al. 1985; Goldmuntz et al. 1986; Dimitrijević et al. 2007). Interestingly, modulating the noradrenergic system can attenuate EAE development. These findings are particularly noteworthy, since a prolonged exposure of cells to NE downregulates and desensitizes ARs, particularly in vitro (Stofkova et al. 2019; Akinaga et al. 2013; Heck and Bylund 1997; Leeb-Lundberg et al. 1987).

## Gateway reflexes: unique neural circuits that regulate initiation and manifestation of autoimmune diseases via alterations in CNS endothelial cells

### Gravity gateway reflex

The posture of the body in the gravitational environment of Earth requires steady muscular tonus to provide accurate body configuration and verticality. Mechanically, this process involves the constant neural activation of anti-gravity muscles, which is controlled by a typical self-regulatory feedback mechanism. For example, coping with gravity to maintain sitting or standing upright mostly does not need any voluntary attention. The anti-gravity muscles basically act through a monosynaptic stretch reflex consisting of an afferent proprioceptive sensory nerve fiber from the muscle spindle, which has its body in the dorsal root ganglia (DRG) of the spinal cord and makes synapses in the ventral spinal cord with alpha motor neurons that innervate the same muscle (Rossi-Durand 2006).

**Fig. 1:**
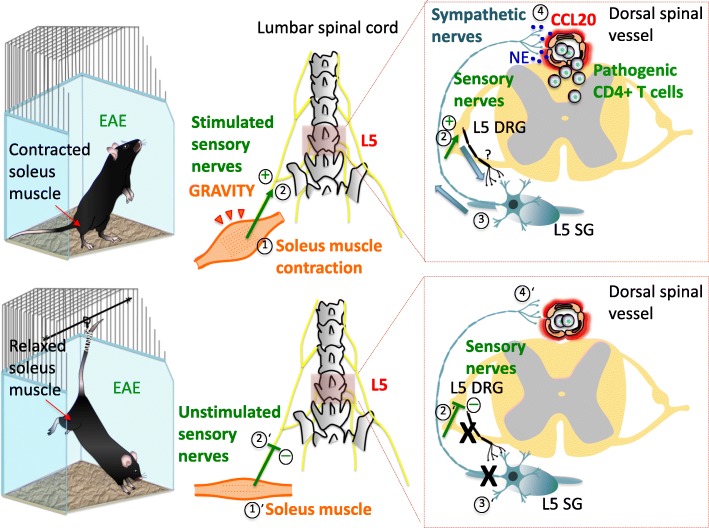
Gravity gateway reflex. In free-moving mouse (upper panel) with adoptive-transfer EAE, pathogenic CD4+ T cells infiltrate the spinal cord parenchyma at the fifth lumbar (L5) level. This process is triggered by soleus muscle contraction (1) that counteracts gravity to maintain stability and body posture. Stimulated sensory nerves arising from the soleus muscle (2) and entering the L5 spinal cord through dorsal root stimulate sympathetic nerves (3) at the L5 spinal cord level via an unidentified neural circuit (?). This leads to chemokine expression including CCL20 in regional dorsal vessels of the spinal cord (4), establishing an immune cell gateway for migration of pathogenic CD4+ T cells across the BSCB. In tail-suspended mouse (lower panel) with adoptive-transfer EAE, soleus muscle relief from gravitational force (1’) prevents regional activation of sensory- (2’) and sympathetic-nerves (3’), and infiltration of pathogenic CD4+ T cells in the L5 spinal cord (4’). Abbreviations: EAE, experimental autoimmune encephalomyelitis; DRG, dorsal root ganglion; SG, sympathetic ganglion; NE, norepinephrine; BSCB, blood-spinal cord barrier

Since the stretch reflexes of the anti-gravity muscles are constantly active in everyday activities depending upon posture, it is not surprising that this neural activity has an impact on regional spinal cord blood flow and NVU dynamics (Marcus et al. 1977). In healthy subjects, such neural activity does not breach the BSCB. However, in the course of CNS autoimmune inflammation in the presence of pathogenic CD4+ T cells, such as EAE, neural impulses from the muscle spindle sensory neurons to the spinal cord can dramatically alter the properties of BSCB endothelial cells (Arima et al. 2012). Specifically, we observed that this neural activity, particularly noradrenergic activity, is able to induce a pro-inflammatory phenotype of endothelial cells around the spinal cord via NF-κB activity. Thus, endothelial cells upregulate NF-κB targets such as chemokines and IL-6 to accumulate pathogenic CD4+ T cells in a manner dependent on the antigen specificity. Pathogenic CD4+ T cells express excessive amounts of cytokines including IL-17, IFNγ, IL-6, and TNFα to simultaneously activate two transcription factors, NF-κB and STAT3, in the regional endothelial cells, resulting in more chemokine and IL-6 expression, a phenomenon known as the inflammation amplifier (originally the IL-6 amplifier) (Ogura et al. 2008; Atsumi et al. 2014; Nakagawa et al. 2015; Murakami et al. 2019). Activation of the inflammation amplifier at regional blood vessel sites increases the BSCB permeability, allowing for the accumulation of various immune cells in the 5th lumbar region (L5) of the CNS. Interestingly, in this model system, systemic inflammation is minimal and thereby has no impact on the systemic endothelial permeability during the initial phase of the disease development (Arima et al. 2012). Because the signals transmitted to endothelial cells by this neural pathway depend on the stretch reflex arc of anti-gravity muscles and create gateways for immune cells to cross the BSCB, we named this mechanism the gravity gateway reflex (Pavlov et al. 2018; Tanaka et al. 2017b; Kamimura et al. 2018a, b)(Fig. 1).

The stretch reflex plays a physiological role to counterbalance gravity under a homeostatic principle. Because the gravity gateway reflex is one of the responses of the stretch reflex against gravity, the gravity gateway reflex should exist under homeostatic conditions on Earth. In other words, the gravity-triggered reflex arc should have physiological roles for CNS homeostasis. We hypothesized that the gravity-mediated L5 gateway establishes an immune surveillance mechanism for pathogen infection and tumor development in the CNS to keep a certain level of immune cells in the CNS.

To extend our understanding of the nature of the neural stimuli underlying changes in barrier endothelial cells in response to sensory nerve activations, we performed a series of experiments in mice with pathogenic CD4+ T cell transfer: 1) tail suspension experiments, in which the hind legs (soleus muscle) are freed from gravity stimulation (Morey-Holton and Globus 2002) and electrostimulated to test the relative contribution of peripheral neurons to the gravity gateway reflex, and 2) the inhibition of EPI signaling using α_1_-AR or β_1_-AR antagonists to investigate the role of sympathetic neurons in the gravity gateway reflex.

The tail suspension model employed in the first series, also known as the hindlimb-unloading model, is a well-documented model of microgravity that mimics neuromuscular alterations during spaceflights. Since the soles of the animal hind limbs are not in the contact with the ground, there is no stimulus of the sensory fibers in anti-gravity hind limb muscles, such as the soleus, to activate the postural reflexes required for the upright stance (De-Doncker et al. 2005; Kawano et al. 2007; Ohira et al. 2002). We found that this model with pathogenic CD4+ T cell transfer caused reduced NF-κB and STAT3 activation along with suppressed CCL20 expression and immune cell infiltration in the dorsal blood vessels of the fifth lumbar cord (L5). Interestingly, since these suspended EAE mice were in a handstand position, CCL20 expression and pathogenic CD4+ T cell accumulation increased in the cervical spinal cord blood vessels. Consistent with these findings, c-fos expression was downregulated in L5 DRG, but upregulated in cervical spinal cord DRG.

It is possible that the hindlimb-unloading model itself is a stressor. However, one week of tail suspension did not cause any difference in the absolute weight of the adrenals of suspended and control animals (Ohira et al. 2002). Thus, the stress induced by this model is minimal. It is also possible that the animals can perceive some weight of the limbs, but the postural reflex activity is at least partially prevented by unloading the hind limbs. Importantly, electrostimulation, which mimics gravity responses via neural pathways of the muscles, increased chemokine expression in the dorsal vessels of the L5, which strongly suggests that a specific neural pathway triggered by gravity responses induces chemokine expression in specific vessel sites of the L5 cord.

In the second series, we reasoned that somatic sensory neurons innervating skeletal muscles interact with peripheral sympathetic neurons that innervate specific dorsal vessels of the spinal cord, where immune cells including pathogenic CD4+ T cells accumulate. c-fos expression in the sympathetic ganglia at the L5 spinal cord was significantly increased and correlated with CCL20 expression at the L5 compared to the L1 spinal cord level, where pathogenic CD4+ T cells do not preferentially enter the spinal cord parenchyma. We found that both α_1_-AR or β_1_-AR antagonists could suppress EAE development as well as the CCL20 expression, NF-κB activation, and immune cell infiltration around the L5 dorsal vessels (Arima et al. 2012).

Based on our observations, we concluded that the gravity gateway reflex is a somato-sympathetic reflex induced by proprioceptive stimuli applied to anti-gravity muscles. Though our data indicate that proprioceptive sensory nerve fibers from muscle spindles represent the afferent pathway and sympathetic nerve fibers represent the efferent pathway of the gravity gateway reflex, the location of the reflex center or anatomical connections between the DRG and sympathetic ganglia remain to be elucidated.

### Electric gateway reflex

**Fig. 2:**
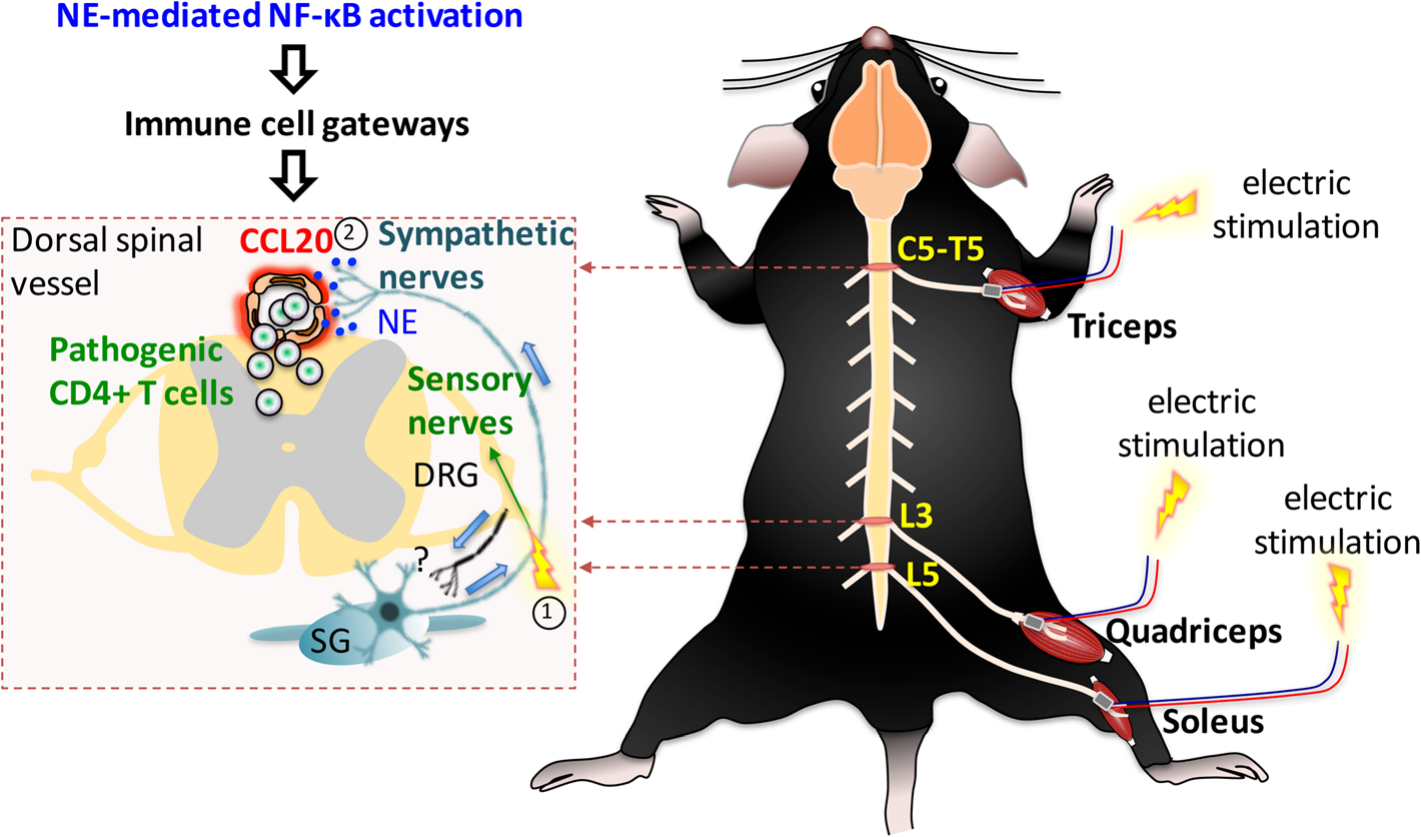
Electronic gateway reflex. Artificial neural activations by weak electric stimulations can induce the gateway reflex at individual blood vessels in the CNS. Electric stimulations to the triceps (1) induce chemokine upregulation at the dorsal vessels of the fifth cervical (C5) to fifth thoracic (T5) spinal cord (2) via the inflammation amplifier, which is mediated by NE secretion. Likewise, electric stimulations to the quadriceps (1) trigger chemokine upregulation at the L3 dorsal vessels (2), whereas the L5 gateway is formed by electric stimulation to the soleus muscles. Abbreviations: DRG, dorsal root ganglion; SG, sympathetic ganglion; NE, norepinephrine

In view of the apparent gateway reflex effect with pathogenic CD4+ T cell infiltration at the L5 dorsal vessels after electrostimulation described above (Arima et al. 2012), we investigated the mechanism mediating this process. An obvious hypothesis was that the stimulation of sensory neurons, which carry information from different muscles via the dorsal root of the spinal cord, would cause the formation of gateways for pathogenic CD4+ T cells through the upregulation of chemokines in the dorsal vessels of different spinal cord levels via a similar NE-NF-κB axis. To test this hypothesis, we applied electrostimulation to the quadriceps and epitrochlearis/triceps brachii in tail suspended mice. Interestingly, we observed an increased expression of chemokines including CCL20 in the dorsal spinal vessels at the level of entry of the respective sensory neurons. Indeed, the stimulation of the quadriceps resulted in CCL20 expression at the 3rd lumbar (L3) dorsal spinal vessels and the stimulation of the epitrochlearis/triceps brachii triggered CCL20 expression between the 5th cervical (C5) and 5th thoracic (T5) dorsal spinal vessels. Moreover, we also found increased c-fos expression in the L3 DRG and the DRG between C5 and T5, suggesting activation of sensory afferent neurons in response to the electrostimulation, since the bodies of these neurons are located in the DRGs (Arima et al. 2012). Together, these findings indicate that muscle electrostimulation can activate a pro-inflammatory response (“electric gateway reflex”, Fig. 2) in the dorsal vessels of the spinal cord, suggesting a possible therapeutic application of electrical muscle stimulation on pathological conditions such as MS. We might have a new delivery method for immune cells to the CNS by the electric gateway reflex, suggesting that the electric gateway reflex could be a form of bioelectronic medicine akin to the inflammatory reflex.

### Pain gateway reflex

Chronic neuropathic pain is one of the most common debilitating symptoms of MS. Many patients with MS usually suffer from pain sensation in multiple areas of the body. The most frequent is central neuropathic (“dysesthetic”) extremity pain, back pain and trigeminal neuralgia (O’Connor et al. 2008). Moreover, MS patients are at a higher risk of developing complex regional pain syndrome (CRPS) than the general population (Schwartzman et al. 2008). In CRPS, even without a definable nerve injury, formerly known as reflex sympathetic dystrophy syndrome (Stanton-Hicks et al. 1995), sympathetically mediated pain often occurs and can be relieved by α-AR antagonists or a sympathetic ganglion block (Campbell and Meyer 2006). Studies using primate models of neuropathic pain induced by spinal nerve ligation indicate that the mechanisms of the sympathetically mediated pain involve an adrenergic sensitization of nociceptors. Specifically, it has been shown that sympathetic nerve fibers sprout into superficial areas of the skin, where they come into close contact with sensory nociceptive terminals that express α_1_-ARs. In this way, NE released from sympathetic terminals activates nociceptive terminals to produce a nociceptor response (Campbell and Meyer 2006).

Given our findings that the function of the gravity gateway reflex is related to the activation of peripheral sympathetic nerves, we hypothesized that sympathetically mediated pain sensation in mice plays a role in the development of transfer EAE. Indeed, our study suggests a bidirectional relationship between pain and activity of the sympathetic nervous system (Arima et al. 2015). Not only can sympathetic nerve impulses trigger pain sensation, but sympathetic responses can be driven by pain (Campbell and Meyer 2006; Burton et al. 2016). This connection is particularly important in the situation of disease remission, where pain-mediated sympathetic responses may reactivate quiescent neuroinflammation. We found that neuropathic pain in EAE-recovered mice triggered relapse via sensory nociceptive-sympathetic coupling in a similar manner as the gravity gateway reflex (Arima et al. 2015). In that study, we employed a chronic constriction injury (CCI) model of neuropathic pain, which was induced by a loose ligation of the infraorbital sensory nerve from the maxillary division of the trigeminal nerve. The trigeminal neuropathic pain was proposed to require activation of the anterior cingulate cortex (ACC) for the development of pain-associated behavior and thalamic sensory neuron activity (Moon et al. 2017). The ACC is a key cortical structure important not only for acute pain but also for neuropathic pain processing and perception (Vogt 2005; Moon et al. 2017). A positive correlation between the activity of the ACC and pain-induced sympathetic vasoconstrictor reflexes as well as sympathetic responses that occur during pain anticipation have been reported in humans, suggesting functional links between the ACC, central sympathetic pathways and pain experience (Seifert et al. 2013). This involvement of the ACC in the regulation of autonomic processes can be attributed to its anatomical connections with brainstem structures (Critchley et al. 2003; Luu and Posner 2003).

**Fig. 3:**
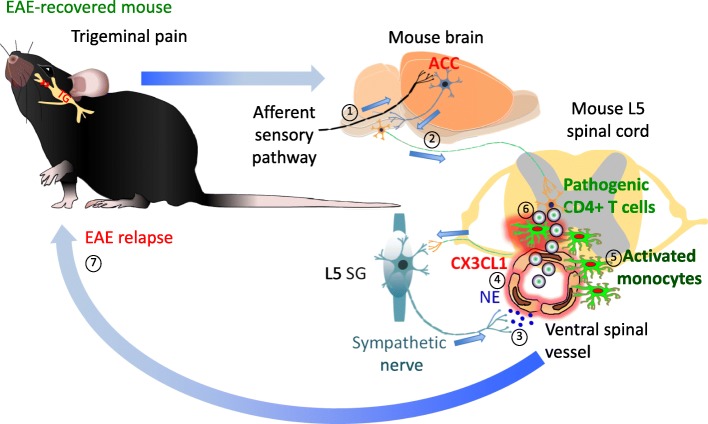
Pain gateway reflex. In a transfer EAE-recovered mouse, induction of trigeminal pain by ligation of the infraorbital sensory nerve causes EAE relapse. In this condition, pathway carrying nociceptive information activates the ACC (1), a pain-processing area of the brain, which in turn stimulates sympathetic centers (2) and their neural output to the ventral spinal vessels (3). NE release around these vessels induces chemokine CX3CL1 expression from activated monocytes and endothelial cells (4). CX3CL1 recruits these activated monocytes around the ventral vessels in an autocrine/paracrine manner (5). Because the L5 spinal cord contains a high number of activated monocytes in EAE-recovered mice, the L5 region is most affected in terms of the accumulation of activated monocytes. Activated monocytes present MOG antigens to reactivate circulating pathogenic CD4+ T cells (6), thus causing their accumulation in the spinal cord and EAE relapse (7). Abbreviations: EAE, experimental autoimmune encephalomyelitis; ACC, anterior cingulate cortex; NE, norepinephrine; MOG, myelin oligodendrocyte glycoprotein; TG, trigeminal ganglion

CCI and capsicin stimulation both activate the ACC in EAE mice with remission (Arima et al. 2015). This activation is followed by increased neuronal activity in sympathetic ganglia at the whole spinal cord level and development of EAE relapse due to the presence of major histocompatibility complex class II+ (MHC II+) CD11b + cells in the L5 cord. In EAE-recovered animals, we have observed an increased number of MHC class II + CD11b + cells with MOG-antigen presentation ability and of peripheral origin around the L5 spinal cord (Arima et al. 2015). Subsequently, after pain induction, many immune cells including MOG-specific CD4 + T cells have been found to infiltrate parenchyma of the L5 spinal cord following the accumulation of MHC class II + CD11b + cells at two ventral vessel sites of the spinal cord. These findings led us to examine the role of MHC class II + CD11b + cells in pain-induced EAE relapse. We found that in response to NE from sympathetic activation around the vessels, these cells expressed a chemokine, CX3CL1, which accumulates the same cells through β_1_- and β_2_-mediated signaling at the vessel sites. Additionally, by directly injecting an antagonist of NMDA receptors, MK801, or agonist of NMDA receptors, L-Homocysteic acid, to the ACC, we found the accumulation of MHC class II+ CD11b + cells at the ventral vessels in the L5 cord is at least in part dependent on ACC activity. We then analyzed the involvement of downstream sympathetic pathways on the infiltration of MHC class II + CD11b + cells around the ventral vessels of the spinal cord using the β_1_-AR antagonist atenolol or 6-OHDA-mediated sympathectomy. Both strategies led to systemic suppression of the sympathetic nervous system and protected the L5 spinal cord area from MHC class II + CD11b + cell infiltration and EAE relapse. Our analyses of the afferent sensory pathway using various pharmacological or genetic manipulations showed that activated neurons processing nociceptive information from the maxillary region to the brain express TRPV1 and Nav1.8 (Arima et al. 2015). Consistent with the importance of the TRPV1+ sensory pathway for EAE relapse is the dependency of chronic pain sensation on the pathway (Huang et al. 2019). Thus, neuropathic pain-mediated activation of the ACC with subsequent stimulation of the sympathetic nerves distributed around the ventral vessels of spinal cords exacerbates quiescent neuroinflammation in the spinal cord by opening gateways for pathogenic CD4 + T cells to enter the CNS in the presence of MHC class II + CD11b + peripheral-derived activated monocytes. Considering the similar nature of this mechanism to the gravity gateway reflex, we named it the “pain gateway reflex” (Fig. 3) (Arima et al. 2015; Tanaka et al. 2017b; Kamimura et al. 2018a, b). The proposed arc of this reflex is composed of sensory nociceptive neurons expressing TRPV1 and Nav1.8 as the afferent pathway, the ACC with its connections to autonomic nuclei in the brainstem as the center, and sympathetic neurons as the efferent pathway. In the pain gateway reflex, both MHC class II + CD11b + activated monocytes and endothelial cells of spinal cord ventral vessels appear to be the initial effector cells.

### Stress gateway reflex

The hypothalamus plays a key role in orchestrating endocrine, autonomic, and behavioral responses to stress. With regard to autonomic responses, communications between the hypothalamus and autonomic centers in the brainstem and spinal cord are bidirectional. Moreover, ascending projections from the autonomic nuclei to the hypothalamus and descending projections in the opposite direction may use the same pathways. Most viscerosensory signals, which are related to autonomic functions such as cardiovascular, respiratory, osmolarity, gastric motility, and food intake, reach the hypothalamus mainly through the nucleus of the solitary tract (NTS), which receives signals from the vagus nerve and glossopharyngeal nerve. Somatosensory signals, including nociception, project to the hypothalamus as well. Especially neurons in the lateral hypothalamus at the level of the paraventricular nucleus (PVN) appear to be crucial, as they significantly increase c-fos expression after painful stimuli (Palkovits 1999; Benarroch 2005). The lateral hypothalamus is also suggested to relay viscero- and somatosensory signals to other hypothalamic nuclei and limbic nuclei, and vice versa. In this way, the lateral hypothalamus serves as an integration center in long-loop autonomic reflexes for mediating cardiovascular, behavioral, and endocrine responses elicited from the insular cortex, limbic areas, and medial hypothalamic nuclei, i.e. the arcuate nucleus (ARC), ventral medial nucleus (VMN), dorsal medial nucleus (DMN), anterior hypothalamic area (AHP), and PVN (Palkovits 1999).

The gateway reflexes use the same neuronal pathways as reflexes activated by natural physiological stimuli (e.g. gravitational and nociceptive) to breach the BSCB and exacerbate EAE outcomes. However, if gateway reflexes utilize pathways of vital autonomic reflexes like cardiovascular or gastrointenstinal reflexes, they may not exacerbate EAE, but they could compromise vital functions to the extent of causing sudden death (Arima et al. 2017). We employed two stress conditions including a light sleep and a hostile environment, both of which can be experienced in normal social life. Importantly, these conditions cause discomfort, but they are not life-threatening (Arima et al. 2017). Nevertheless, in mice with transferred pathogenic CD4+ T cells under these chronic stress conditions, we observed severe gastrointestinal inflammation and heart failure including catabolism of vital tissues without the presence of typical EAE symptoms such as tail or leg paralysis. Importantly, in these mice, pathogenic CD4+ T cells and MHC class II+ cells accumulated at two symmetrical brain vessel sites of the boundary region of the third ventricle (3 V), thalamus, and dentate gyrus (DG), but not in the L5 spinal cord, which is where they accumulated in unstressed EAE animals undergoing the gravity gateway reflex. We hypothesized that the infiltration of immune cells at the specific sites of the brain might be due to upregulated chemokine expressions in vascular endothelial cells of regional vessels followed by the formation of gateways for immune cells to pass through the BBB. To assess this possibility, we tested several neutralizing antibodies against chemokines known to recruit pathogenic CD4+ T cells and MHC class II+ antigen presenting cells. An anti-CCL5 antibody was shown to prevent the mortality of EAE mice under chronic stress. Consistent with this observation, we found that chronic stress could induce CCL5 expression at the specific vessels as well as activate PVN noradrenergic neurons. Moreover, many neurons in the DMH (dorsomedial hypothalamic nucleus) and AHP were activated in mice with stress plus pathogenic CD4+ T cells compared to mice with stress or pathogenic CD4+ T cells alone. Neural tracing experiments showed direct neural connections between the specific vessels and neurons in the PVN or DMH/AHP. To study the functional importance of noradrenergic neurons at the vessels, we performed chemical sympathectomy. The microinjection of 6-hydroxydopamine at the vessels, which depleted tyrosine hydroxylase sympathetic neurons and phospho-CREB signals, suppressed the microinflammation development and the fatal outcome in mice with stress plus pathogenic CD4+ T cells. To study the functional importance of neurons in the PVN and DMH/AHP, we performed electric cauterization of the brain regions, finding the fatal phenotype was a suppressed in mice with stress plus pathogenic CD4+ T cells (Arima et al. 2017). We are also planning to perform optogenetic and chemogenetic experiments to clarify the functions of the neural connections and to identify neural markers.

**Fig. 4:**
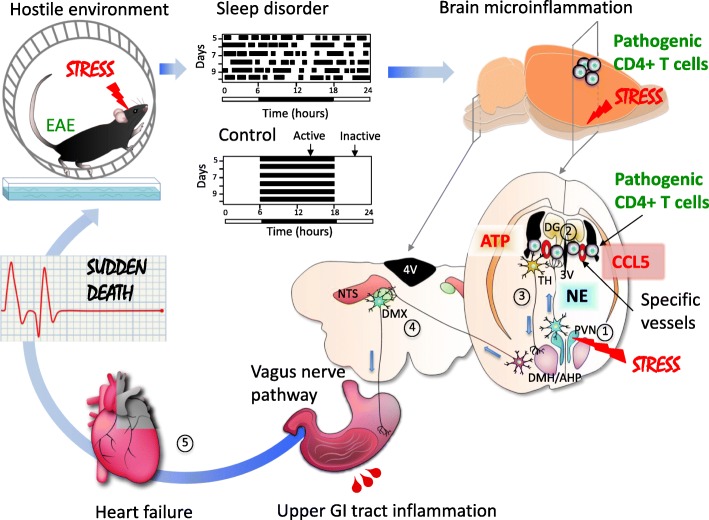
Stress gateway reflex. Hostile environment induced by a specific cage with water and a free rotation wheel causes chronic stress associated with sleep disorder. In a mouse with adoptive-transfer EAE, this chronic stress stimulates noradrenergic neurons in the PVN (1), which then project to bilateral specific vessels surrounded by the third ventricle (3V), dentate gyrus (DG) and thalamus (TH) (2) to upregulate chemokines including CCL5 and recruit pathogenic CD4+ T cells plus MHC class II+ monocytes. The resulted microinflammation activates a neural pathway involving the DMH/AHP and DMX via ATP production (3). Hyperactivation of the vagus pathway particularly distributed the upper gastrointestinal (GI) tract causes a severe damage in the upper GI tract followed by a heart failure with sudden death (5). Abbreviations: AHP, anterior hypothalamic area; EAE, experimental autoimmune encephalomyelitis; PVN, paraventricular nucleus of the hypothalamus; DMH, dorsomedial nucleus of the hypothalamus; DMX, dorsal motor nucleus of the vagus nerve; NTS, nucleus tractus solitarii; 4V, fourth ventricle

Importantly, activation of the DMH/AHP was associated with descending ATPergic projections arising from the specific inflamed brain vessel sites in the presence of pathogenic CD4+ T cells and MHC class II+ antigen presenting cells. Stimulation of this pathway was associated with the sudden death of EAE mice exposed to stress conditions, because the intracranial injection of A438079, an ATP P2RX7 receptor antagonist, at the specific vessels could suppress the DMH/AHP activation and the mortality caused by severe gastrointestinal bleeding and heart failure (Fig. 4) (Arima et al. 2017). ATP is a fast excitatory neurotransmitter, and its function as a cotransmitter in central and peripheral noradrenergic neurons is well established (Zimmermann 1994; Burnstock 2006). Furthermore, ATP as a neurotransmitter was reported to act on postsynaptic P2X receptors to modulate the excitability of DMH/AHP neurons (Matsumoto et al. 2004; Burnstock 2007). Activation of the DMH/AHP has been shown to stimulate intestinal motility, heart rate and blood pressure through vagal cholinergic, nonvagal cholinergic and noncholinergic pathways (Greenwood and DiMicco 1995). We found that activation of the vagal motor nerves most likely mediated by a DMH–DMX (dorsal motor vagal nucleus) pathway was critical for the development of the fatal gastrointestinal failure under stress conditions in EAE mice, because vagotomy suppressed the stress-EAE phenotype with sudden death. We also found cholinergic activation in epithelial cells in the upper gastrointestinal tract was affected. Additionally, enhanced activation of the NTS, which receives afferent inputs from the vagus nerve, indicated increased vagal sensory signaling to the brain in mice under stress conditions and pathogenic CD4+ T cell transfer (Arima et al. 2017). These vagal afferent inputs to the NTS likely arise from baroreceptors, since the baroreflex is chronically activated in conditions of high blood pressure and heart rate as a compensatory response (Lohmeier and Iliescu 2015). The increased neural activity associated with these autonomic long-loop reflexes controlling blood pressure during chronic stress might explain the activation of noradrenergic PVN neurons projecting to specific vessels in the area of the 3 V, thalamus, and DG, resulting in BBB breakdown and the formation of immune cell gateways. Microinflammation in the boundary region of the 3 V, thalamus, and DG appears to be critical, given the lethal effect of the intracranial injection of pro-inflammatory cytokines such as IL-6 plus IL-17A, IFNγ plus IL-17A, or pathogenic CD4+ T cells plus antigen-presenting cells to this brain area. Importantly, none of stress, the transfer of pathogenic CD4+ T cells alone, or the combination of stress with the transfer of activated CD4+ T cells directed against non-CNS antigens such as ovalbumin or interphotoreceptor retinoid-binding protein (IRBP) were able to cause the fatal phenotype (Arima et al. 2017).

Circulating EPI could affect the phenotype of endothelial cells in the brain, but because it is also a strong vasoconstrictive agent it can also prevent gastrointestinal bleeding. In fact, endoscopic injection of EPI is widely used in clinical practice in patients with actively bleeding ulcers (Lin et al. 1999; Liou et al. 2006; Vergara et al. 2014). Therefore, it is important to investigate whether peripheral levels of catecholamines, which are released from the adrenals in response to chronic stress, contribute to the gastrointestinal failure. We performed experiments using two antagonists of the corticosteroid receptor, mifepristone and guggulsterone, on mice in a stress condition after the microinjection of cytokines at specific vessels (Arima et al. 2017). The antagonists had no effect on the cytokine-mediated fatal phenotype, which suggests the hypothalamic–pituitary–adrenal axis has a minimum role.

We named this autonomic long-loop reflex circuit exploited by stress-mediated brain microinflammation the “stress gateway reflex”. To summarize our current understanding of this fatal gateway reflex, our findings support the proposal that overactivation of central noradrenergic signaling induced by stress accounts for a pro-inflammatory phenotype of the BBB endothelium at specific brain vessel sites characterized by increased CCL5 expression and triggers the infiltration of pathogenic CD4+ T cells and activated MHC class II+ monocytes. The microinflammation induced by pathogenic CD4+ T cell activation stimulates fast excitatory ATPergic neurons descending the DMH/AHP, which in turn activates efferent (motor) vagal outflow to the upper gastrointestinal tract. Constant stimulation of the gateway reflex under chronic stress leads to gastric inflammation, gastrointestinal bleeding, hyperkalemia, and eventually heart failure (Arima et al. 2017; Kamimura et al. 2018a, b).

Our findings are in contrast with previous studies that suggested an anti-inflammatory role of the vagus nerve in murine and rat colitis models (Ghia et al. 2006, 2007; Meregnani et al. 2011) or murine postoperative ileus (de Jonge et al. 2005). These studies are in parallel with promising results of bioelectronic medicine targeting the vagus nerve of patients with rheumatoid arthritis, inflammatory bowel disease, and Crohn’s disease (Merrill et al. 2006; Schwartz et al. 2008; González et al. 2019; Engineer et al. 2019; Koopman et al. 2016; Bonaz et al. 2016). The discrepancy is most likely due to the fact that the activated vagus fibers are different. It is possible that stress gateway reflex activates only vagus fibers distributed on the upper gastrointestinal tract, while bioelectronic medicine stimulates almost all vagus nerves. Consistent with this hypothesis, vagus nerves with mechanosensor PIEZO+ alone are critical for the baroreceptor reflex (Zeng et al. 2018), while P2ry1 and Npy2r vagal sensory neuron types evoke distinct effects on breathing (Chang et al. 2015). Moreover, vagal GPR65 neurons control gut motility, and GLP1R neurons detect stomach and intestine stretch (Williams et al. 2016). Therefore, it is possible that there exist specific functional markers of immunosuppressive vagus fibers and/or vagus fibers that distribute or function only on the upper gastrointestinal tract. Further studies are required to gain broader insight of the effect of vagus nerve stimulation on autoimmune diseases, particularly those associated with chronic stress and CNS inflammation.

The suggested arcs of the stress gateway reflex consist of two branches. The first branch is composed of (1) interoceptors distributed in blood vessels and the heart, (2) an afferent pathway represented by sensory vagal nerves transmitting information from the interoceptors to (3) the NTS and PVN centers in the brain, (4) an efferent pathway represented by noradrenergic neurons ascending from the PVN, and (5) endothelial cells of the specific vessel sites at the boundary area of the 3 V, thalamus, and DG that function as effectors. The second branch of the stress gateway reflex starts with (1) afferent ATP-producing neurons at the boundary area of the 3 V, thalamus, and DG, which are activated by microinflammatory stimuli projecting to (2) the reflex center in the DMH/AHP, which then transmits signals to the DMX to activate (3) efferent vagal nerves, resulting in increased gastrointestinal secretory and motor functions mediated by (4) local gastrointestinal cells.

### Light gateway reflex

When bright light enters the eyes, the pupils decrease in size to reduce the amount of light reaching the retina. On the contrary, under dim light, the pupils enlarge to allow as much light as possible to reach the retina. This mechanism, known as the pupillary light reflex, is a result of interactions between neurons and iris smooth muscle cells. Under photopic light condition, photoreceptive pigments in the photoreceptors and intrinsically photosensitive retinal ganglion cells (ipRGCs) absorb visible light photons and convert this light energy into nerve impulses. These impulses are then transmitted via the optic nerve pathway to the pupillary light reflex center in the midbrain: Edinger­Westphal nuclei. Efferent projections from these nuclei travel to the ciliary ganglion, where parasympathetic post­ganglionic neurons of the oculomotor nerve extend to innervate the pupillary sphincter muscles leading to pupillary constriction. In mesopic and scotopic light conditions, the pupil enlargement is under sympathetic influence on the iris dilator muscle and consists of a three-neuron arc extending from the retinohypothalamic tract through the ciliospinal center of Budge in the intermediolateral gray column of the spinal cord at C8–T1 to the superior cervical ganglion (SCG), from which sympathetic post­ganglionic neurons travel to and innervate the iris dilator muscle to cause pupil dilation (McDougal and Gamlin 2015; Hall and Chilcott 2018; Belliveau and Dossani 2019).

Using an animal model of posterior autoimmune uveitis, experimental autoimmune uveoretinitis (EAU), we investigated whether the pupillary light reflex pathway influences the infiltration of immune cells into the retina. To avoid the disruption of circadian rhythms, we stimulated mice with mesopic light of 2 lx or photopic light of 230 lx only in the light phase of the day. In the dark phase, all mice were exposed to 0 lx condition. Furthermore, these light interventions were applied only four days, starting from the first appearance of CD4+ T cells in the eyes after the onset of the clinical phase of EAU (Stofkova et al. 2019).

We observed that photopic and mesopic light can control retinal vascular endothelial cells via neural regulation. Indeed, mice housed under photopic light during EAU development exhibited a downregulation of NF-κB and STAT3 activity in the retinal endothelium, decreased gene expressions of various chemokines and cytokines that attract and/or activate immune cells in the retina, and reduced EAU development compared to EAU mice housed under mesopic light. Consistently, EAU mice exposed to photopic light showed lower numbers of CD4+, CD8+ and CD11b + cells in the retina and around the central retinal vessels within the optic nerve head in comparison to EAU mice exposed to mesopic light.

**Fig. 5:**
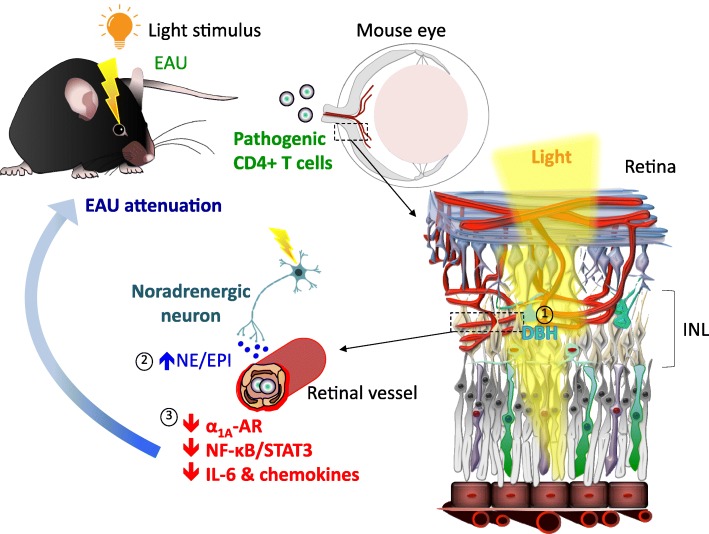
Light gateway reflex. Exposure to photopic light in actively induced EAU attenuates retinal inflammation. This process is mediated through light-induced expression of DBH, an enzyme essential for the synthesis of NE and EPI, by retinal neurons located in the INL (1). The increased NE and EPI levels down-regulate retinal α_1A_AR expression, which is followed by reduced NF-κB and STAT3 activation and decreased chemokine and IL-6 expressions in the retina (2)(3). This protects the BRB integrity and suppresses recruitment of immune cells including IRBP-specific pathogenic CD4+ T cells in the retina. Abbreviations: EAU, experimental autoimmune uveoretinitis; DBH, dopamine beta-hydroxylase; INL, inner nuclear layer; NE, norepinephrine, EPI: epinephrine; α_1A_AR, alpha-1A adrenoceptor; BRB, blood-retina barrier; IRBP, interphotoreceptor retinoid-binding protein

Given our previous data demonstrating the involvement of sympathetic ganglia in gravity-, electric-, and pain-gateway reflexes and the fact that the SCG is physiologically activated under mesopic light and inhibited under photopic light by the pupillary light reflex, we hypothesized that changes in the retinal endothelium observed in EAU mice housed under different light intensities are related to the activity of sympathetic neurons from the SCG. To test this hypothesis, we performed unilateral surgical ablation of the SCG (SCG-X) to eliminate sympathetic inputs to the eye, and we immunized mice with interphotoreceptor retinoid-binding protein peptide 1–20 (IRBP_1–20_) to induce EAU. However, mesopic light- and photopic light-exposed mice with SCG-X developed EAU in both eyes without significant lateralization of immune cell infiltrates to either eye, suggesting that peripheral sympathetic nerves do not mediate the phenotype of the BRB endothelium during EAU (Fig. 5) (Stofkova et al. 2019). This finding is consistent with the fact that retinal vessels receive minimal sympathetic innervation (McDougal and Gamlin 2015). Since retinal neurons, including amacrine cells and horizontal cells have been shown to produce NE and EPI (Park et al. 1988; Hadjiconstantinou et al. 1983; Ishimoto et al. 1989; Chen et al. 1999), we assessed NE and EPI levels in the eyes in response to photopic and mesopic light in EAU mice. We found a significant increase in NE and EPI levels in the eyes, but not in the serum of mice exposed to photopic light when compared to mesopic light. In addition, we also observed an upregulation of dopamine β-hydroxylase (DBH), an enzyme necessary for NE and EPI synthesis, in the inner nuclear layer (INL) of the retina, which is composed of amacrine, horizontal and bipolar cells. Simultaneously, we also detected the downregulation of α_1_-AR expression in retinal endothelial cells in EAU mice exposed to photopic light. We assume that this downregulation is due to high NE and EPI levels in the eyes, since AR ligands downregulate AR expression (Stofkova et al. 2019; Akinaga et al. 2013; Heck and Bylund 1997; Leeb-Lundberg et al. 1987). Consistent with this effect, pharmacological or genetic inhibition of α_1_-AR or α_1A_-AR reduced the activity of noradrenergic signaling to suppress pro-inflammatory cytokine/chemokine expressions in the retina and EAU development under mesopic light condition (Stofkova et al. 2019).

We therefore concluded that NE/EPI-producing neurons after photopic light treatment in the retina interact with retinal endothelial cells to inhibit the formation of immune cell gateways during EAU. Since this mechanism, like the gateway reflexes observed in EAE, requires the neural stimulation of effector barrier endothelial cells, we named it the “light gateway reflex” (Fig. 5). However, unlike the pro-inflammatory gateway reflexes associated with EAE, the light gateway reflex is an anti-inflammatory reflex and seems to be integrated entirely within the retina independently of the central or peripheral nervous system. Thus, we found an unprecedented mechanism by which retinal neural activity mediated by light can regulate the local homeostasis and defend the local tissue against invading immune cells without utilizing other ocular reflexes related to SCG activity.

## Conclusion

We here shed light on new aspects of neuroinflammation and how neural activity can modulate the formation of immune cell gateways to initiate and progress the development of autoimmune diseases of the CNS and retina. The principle of this process is called the gateway reflex, which can be defined as neural circuits regulating the entry of immune cells to the CNS and retina by modulating the barrier phenotype of vascular endothelial cells. We have characterized four pro-inflammatory gateway reflexes that promote the development and outcome of experimental models of MS: gravity-, electric-, pain-, and stress-gateway reflexes. All these reflexes should be utilized as neural pathways of physiological reflexes that serve to keep body homeostasis, particularly in the absence of autoreactive CD4+ T cells, which are known to increase with age and/or infection. Despite the reflexes using distinct viscero- and somatosensory stimuli and afferent pathways for their activation, the efferent pathway resulting in the pro-inflammatory phenotype of endothelial cells is mainly noradrenergic. We also characterized one anti-inflammatory reflex, the light gateway reflex, which protects against the development of an experimental model of posterior autoimmune uveitis and maintains the anti-inflammatory phenotype of the BRB endothelium. The light gateway reflex is unique in that it involves retinal neural circuits to prevent BRB breakdown, and the anti-inflammatory response in this reflex is mediated by the inhibition of noradrenergic signaling in retinal vascular endothelial cells. The molecular mechanism of the light gateway reflex could be a general mechanism that suppresses the formation of immune cell gateways seen in other gateway reflexes, all of which are associated with the transient development of diseases. We could just easily find a negative feedback mechanism in the EAU system, because many neural cells in retina tissues make the responses drastically.

It is reasonable to speculate that regional neural activity at specific vessels can modulate the development of other autoimmune diseases beyond those affecting nervous tissues. An example from clinical research suggesting the importance of neural activity in autoimmune diseases is the sparing effect of hemiplegia in several rheumatoid diseases, particularly in patients with rheumatoid arthritis, psoriatic arthritis, polymyalgia rheumatica or systemic sclerosis (Ughi et al. 2015). In these rheumatic patients, paretic limbs have been shown to exhibit a marked remission of arthritic signs, including diminished erosive changes and decreased skin temperature (Keyszer et al. 2004), suggesting that a loss of neural activity can protect against rheumatoid inflammation. Thus, the next crucial step is to elucidate whether the concept of the gateway reflex is a fundamental phenomenon that also plays a role in the recruitment of immune cells to other tissues in the body.

## Data Availability

Data sharing not applicable to this article.

## Abbreviations

3 V: Third ventricle
ACC: Anterior cingulate cortex
AHP: Anterior hypothalamic area
AR: Adrenoceptor
BBB: Blood-brain barrier
BRB: Blood-retina barrier
BSCB: Blood-spinal cord barrier
CCI: Chronic constriction injury
CNS: Central nervous system
CRPS: Complex regional pain syndrome
CSF: Cerebrospinal fluid
DG: Dentate gyrus
DMH: Dorsomedial hypothalamic nucleus
DMX: Dorsal motor vagal nucleus
DRG: Dorsal root ganglia
E: Embryonic day
EAE: Experimental autoimmune encephalomyelitis
EAU: Experimental autoimmune uveoretinitis
EPI: Epinephrine
GGTP: Gamma-glutamyltranspeptidase
INL: Inner nuclear layer
ipRGCs: Intrinsically photosensitive retinal ganglion cells
IRBP: Interphotoreceptor retinoid-binding protein
MHPG: Methoxyhydroxyphenylglycol
MOG: Myelin oligodendrocyte glycoprotein
MS: Multiple sclerosis
NE: Norepinephrine
NTS: Nucleus of the solitary tract
NVU: Neurovascular unit
PVN: Paraventricular nucleus
SCG: Superior cervical ganglion
VSMCs: Vascular smooth muscle cells
